# Soft Tick Relapsing Fever — United States, 2012–2021

**DOI:** 10.15585/mmwr.mm7229a1

**Published:** 2023-07-21

**Authors:** Amy M. Beeson, Anne Kjemtrup, Hanna Oltean, Hannah Schnitzler, Heather Venkat, Irene Ruberto, Natalie Marzec, Devon Cozart, Leslie Tengelsen, Stephen Ladd-Wilson, Hannah Rettler, Bonny Mayes, Kelly Broussard, Ali Garcia, Lisa L. Drake, Elizabeth A. Dietrich, Jeannine Petersen, Alison F. Hinckley, Kiersten J. Kugeler, Grace E. Marx

**Affiliations:** ^1^Division of Vector-Borne Diseases, National Center for Emerging and Zoonotic Infectious Diseases, CDC; ^2^Epidemic Intelligence Service, CDC; ^3^California Department of Public Health; ^4^Washington State Department of Health; ^5^Arizona Department of Health Services; ^6^Career Epidemiology Field Officer Program, CDC; ^7^Colorado Department of Public Health and Environment; ^8^Montana Department of Public Health and Human Services; ^9^Idaho Department of Health and Welfare; ^10^Public Health Division, Oregon Health Authority; ^11^Utah Department of Health and Human Services; ^12^Texas Department of State Health Services; ^13^Nevada Department of Health and Human Services; ^14^New Mexico Department of Health.

**Summary**

**What is already known about this topic?**

Soft tick relapsing fever (STRF) is a rare but serious bacterial disease spread by *Ornithodoros* ticks. In the United States, acquisition of STRF is associated with rustic cabins, camping, and caves.

**What is added by this report?**

During 2012–2021, a total of 251 STRF cases were identified in 11 of 12 states where infection is reportable; 55% of patients were hospitalized, and no deaths occurred. The geographic distribution and seasonal pattern of STRF have remained relatively constant since the 1990s.

**What are the implications for public health practice?**

Persons should avoid rodent-infested structures and rodent habitats, such as caves, in areas where STRF is endemic. Improvements in surveillance, prevention, and diagnosis are needed to prevent STRF-associated morbidity and mortality.

## Abstract

Soft tick relapsing fever (STRF) (also known as tickborne relapsing fever) is a rare infection caused by certain *Borrelia* spirochetes and transmitted to humans by soft-bodied *Ornithodoros* ticks. In the United States, acquisition of STRF is commonly associated with exposure to rustic cabins, camping, and caves. Antibiotic treatment is highly effective for STRF, but without timely treatment, STRF can result in severe complications, including death. No nationally standardized case definition for STRF exists; however, the disease is reportable in 12 states. This report summarizes demographic and clinical information for STRF cases reported during 2012–2021 from states where STRF is reportable. During this period, 251 cases were identified in 11 states. The median annual case count was 24. Most patients with STRF (55%) were hospitalized; no fatalities were reported. The geographic distribution and seasonal pattern of STRF have remained relatively constant since the 1990s. Persons should avoid rodent-infested structures and rodent habitats, such as caves, in areas where STRF is endemic. STRF surveillance, prevention, and control efforts would benefit from a standardized case definition and increased awareness of the disease among the public and clinicians.

## Introduction

*Ornithodoros* ticks usually inhabit rodent nests and burrows. They can live for decades, and once infected with relapsing fever, *Borrelia* spp., can transmit the bacteria to humans throughout their lifetime through brief and painless bites that are often not detected. Soft tick relapsing fever (STRF) (also known as tickborne relapsing fever) is caused by infection with various *Borrelia* spp., each transmitted by a specific *Ornithodoros* species. STRF is endemic in certain areas in Africa, Asia, Europe, and the Americas. In the United States, two *Borrelia* species, *Borrelia hermsii* and *Borrelia turicatae*, have been confirmed to cause STRF in humans. *B. hermsii*, spread by *Ornithodoros hermsi* ticks, is found in mountainous areas of western states at moderate to high elevations and is commonly associated with rustic, rodent-infested cabins. *B. turicatae*, spread by *Ornithodoros turicata*, is found in the south-central United States and is often associated with caves.

The clinical syndrome of STRF in humans includes high fever, which can be accompanied by headache, nausea, myalgias, and arthralgias. The initial illness typically lasts approximately 3 days; if untreated, febrile episodes can recur every 7–10 days for two or more cycles, because of the spirochetes’ unique ability to repeatedly evade a host’s immune system (*1*). Prompt treatment is important to prevent complications; effective antibiotics include doxycycline, beta-lactam antibiotics (e.g., penicillin or ceftriaxone), and azithromycin (*2*). Rare complications of STRF include neurologic and ocular disease, myocarditis, and acute respiratory distress syndrome (*3*). Infection during pregnancy can result in pregnancy loss, transplacental transmission, and neonatal death (*4*–*6*).

## Methods

In 2021, STRF was reportable in 12 states: Arizona, California, Colorado, Idaho, Montana, Nevada, New Mexico, Oregon, Texas, Utah, Washington, and Wyoming.* Seven of these states used a case definition during 2012–2021; definitions differed among states. This summary describes cases that were classified as confirmed, probable, or suspected,^†^ as well as unclassified cases that met specific criteria.^§^ Trends in annual case counts were assessed using linear regression. This activity was reviewed by CDC and was conducted consistent with applicable federal law and CDC policy.^¶^

## Results

During 2012–2021, 251 cases were identified in 11 states. A median of 24 cases were reported from these states per year (range = 15 [2020] to 41 [2014]). No significant change in the number of cases was observed during this period (p = 0.21). The median age of infected persons was 39 years (range = 2–92 years); 60% were male (Table 1). No infected persons were reported to be pregnant. Race and ethnicity data were available for 190 (76%) persons; among these, 93% were non-Hispanic White persons.

**TABLE 1:** Characteristics of patients with soft tick relapsing fever reported in U.S. states — United States, 2012–2021

Characteristic	No. of cases (%)	No. of patients hospitalized/No. with available hospitalization data (%)
**Total**	**251 (100)**	**115/211 (55)**
Sex		
Female	99 (39)	52/87 (60)
Male	151 (60)	63/123 (51)
Other or unknown	1 (<1)	0/1 (—)
**Age group, yrs**		
≤12	40 (16)	16/36 (44)
13–18	21 (8)	7/11 (64)
19–64	152 (61)	72/134 (54)
≥65	37 (15)	20/30 (67)
**Suspected exposure location**		
Texas*	12 (5)	6/12 (50)
Western United States^†^	217 (93)	104/187 (56)
International	4 (2)	2/2 (100)

* The suspected etiology of soft tick relapsing fever for most patients with exposure in Texas is *Borrelia turicatae* based on known pathogen distribution.

^†^ Includes Arizona, California, Colorado, Idaho, Montana, Nevada, New Mexico, Oregon, Utah, and Washington.

Reported case counts varied by state, with four states accounting for >75% of all cases (California [33%], Washington [18%], Colorado [14%], and Oregon [12%]). Other states with reported cases included Arizona (9%), Texas (5%), Idaho (4%), Utah (3%), Montana (1%), Nevada (1%), and New Mexico (<1%). Among the 12 states with mandated reporting, no cases were reported in Wyoming. Among 232 (92%) cases with available data on state of residence and exposure, 33 (14%) occurred in out-of-state visitors; among the 210 (84%) cases for which county of the patient’s exposure was available (Figure), 118 (56%) occurred in out-of-county visitors. Epidemiologic links to other cases were documented in 21% of cases; the largest outbreak (11 cases) occurred in Arizona in 2014 (*7*). Four (2%) cases were attributed to exposures occurring during international travel to Argentina, Canada, Jordan, and Tanzania.

**FIGURE:**
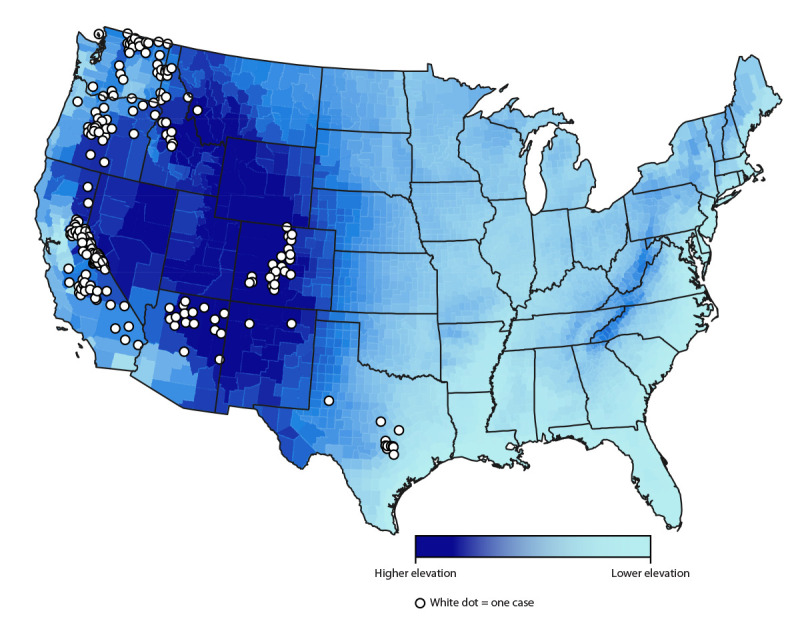
Cases* of soft tick relapsing fever (n = 210),^†^ by county^§^ of exposure — United States, 2012–2021 * The figure does not show exact location of cases because they were arbitrarily placed within the county of exposure. ^†^ Data on county of exposure was not available for all 251 cases included in this report. ^§^ Mean elevation shown per county.

Among 11 reported STRF cases with patient exposures in counties of lower elevations in central Texas, where infections are more likely to be caused by *B. turicatae*, cave exposures were documented in four. Among 217 cases with patient exposures in other western U.S. states, where infections are more likely to be caused by *B. hermsii*, a summer peak was observed, with 154 (71%) cases occurring during June–September. Notable exposures documented among 177 patients in these western states included visits to cabins (131, 74%) and camping (15, 8%).

Some clinical data were provided for 207 (82%) patients with reported STRF (Table 2). Fever was documented in 97% of cases; a median of two distinct febrile episodes (range = 1–9)** was reported among febrile patients. Other commonly reported signs and symptoms included headache (63%), myalgias (59%), chills (54%), and nausea or vomiting (45%). Among 211 patients for whom hospitalization data were available, 115 (55%) were hospitalized, including 44% of 36 children aged ≤12 years and 67% of 30 adults aged ≥65 years (Table 1). No deaths were reported.

**TABLE 2:** Documented signs and symptoms in patients with soft tick relapsing fever (n = 207) — United States, 2012–2021

Sign or symptom	No. (%)* of patients
Fever (at least one episode)	201 (97)
Headache	130 (63)
Myalgias	123 (59)
Chills	112 (54)
Nausea/Vomiting	94 (45)
Sweats	65 (31)
Fatigue/Malaise	65 (31)
Anorexia	63 (30)
Arthralgias	43 (21)
Cough	28 (13)
Altered mental status	24 (12)
Thrombocytopenia	21 (10)
Rash	20 (10)
Photophobia	14 (7)
Neurologic or ocular symptoms^†^	10 (5)
Abdominal pain	9 (4)

* Among persons with available clinical data; patients could have multiple signs or symptoms.

^†^ Reported neurologic or ocular symptoms included uveitis, Bell’s palsy, blurred vision, eye pain, and eye swelling.

Laboratory test data were available for 221 (88%) patients; among these, spirochetes were identified by microscopy of peripheral blood smear in 130 (59%). In addition, relapsing fever *Borrelia* antibodies were detected by serologic testing in 91 (41%) patients, and relapsing fever *Borrelia* DNA was detected by polymerase chain reaction (PCR) testing in 33 (15%).^††^ Relapsing fever spirochetes were cultured in four (2%) cases.

## Discussion

The geographic distribution and seasonal pattern of reported STRF have remained relatively constant since the 1990s (*8*). During 1990–2011, the median annual case count (20) (*8*) was slightly lower than that during 2012–2021 (24); however, the increase from 1990 to 2021 was not statistically significant (p = 0.84). A large proportion of cases continue to occur in nonresident visitors to areas where the disease is endemic (such as vacationers to mountain cabins); cases in returned visitors who live in areas where STRF is not endemic or reportable would be more likely to be missed by clinicians and public health authorities. Though molecular diagnostic testing has become increasingly available in recent years, microscopic examination of peripheral blood smears remains an important diagnostic test; microscopy is most sensitive when performed during febrile episodes because fever is associated with coincident high levels of spirochetemia.

### Limitations

The findings in this report are subject to at least four limitations. First, surveillance for STRF is likely hindered by underrecognition and underdiagnosis; some state health departments have also noted misdiagnosis of STRF as Lyme disease, given that antibodies to STRF-causing *Borrelia* spp. can cross-react with some serologic assays for Lyme disease. The emergence of hard tick relapsing fever (HTRF) caused by *Borrelia miyamotoi* has further complicated accurate diagnosis, particularly in states where both STRF and HTRF might occur, because most serologic and PCR assays do not distinguish between these (*9*). Second, case ascertainment by state health departments is likely limited by underreporting, because states rely primarily on provider reporting.^§§^ Third, case ascertainment is inconsistent across states because of differing or absent case definitions. STRF might also occur in states where it is not currently reportable; a very small number of cases have historically been reported from Oklahoma, Kansas, Ohio, and the U.S. Virgin Islands (*10*). For these reasons, reported cases likely underestimate the true case count. Finally, information on clinical features and exposures was limited to what was obtained by health departments; these data are not collected in all case investigations.

### Implications for Public Health Practice

STRF often occurs in clusters because of common exposures; inhabitants and visitors to a soft tick–infested structure can become infected over multiple decades. Unrecognized or unreported cases are missed opportunities for intervention to prevent future exposures. To reduce STRF incidence in the United States, progress in surveillance, prevention, and disease recognition is needed. A regional standardized case definition has been developed by vectorborne disease epidemiologists in several states with endemic disease; broader adoption of this case definition would enhance STRF surveillance. In addition, residents and visitors to areas where STRF is endemic should be educated about how to prevent soft tick bites (most importantly, avoidance of rodent-infested structures and rodent habitats such as caves) and when to seek medical care. Owners of tick- or rodent-infested cabins should be made aware of recommendations for remediation of these structures.^¶¶^ Clinicians should be aware of the clinical syndrome accompanying STRF, associated exposures, options for diagnostic testing, and public health reporting requirements. Increased awareness of and access to molecular diagnostic testing for symptomatic patients with suspected STRF might improve recognition of cases at different stages of illness. Coordinated improvements in surveillance, prevention, and diagnosis have the potential to prevent morbidity and mortality from STRF in the United States in the next decade.

## Footnotes

* STRF was not consistently reportable in all 12 states during 2012–2021. In Texas, STRF was removed from the reportable diseases list in 2016 and added back in 2021. In Wyoming, relapsing fevers became reportable in 2012.

† Cases were classified as confirmed, probable, or suspected according to each jurisdiction’s case definition.

§ Unclassified cases from states that did not have a case definition in use were reviewed individually. These were included in this case summary if they had laboratory evidence of infection (defined as spirochetes detected on blood smear, relapsing fever *Borrelia* DNA detected by polymerase chain reaction, positive serologic testing for relapsing fever, or isolation of relapsing fever *Borrelia* spirochetes in culture) or if they had a clinical syndrome compatible with relapsing fever together with either exposure to soft tick habitat within 2–18 days of symptom onset or an epidemiologic link to a case with laboratory evidence of infection.

¶ 45 C.F.R. part 46.102(l)(2), 21 C.F.R. part 56; 42 U.S.C. Sect. 241(d); 5 U.S.C. Sect. 552a; 44 U.S.C. Sect. 3501 et seq.

** Relapses were defined as occurrences of fever separated by ≥3 days, although information on timing of fevers was incomplete for some patients.

†† Information on *Borrelia* species was not available, because most laboratory tests do not reliably distinguish between relapsing fever group Borreliae (e.g., between *B. hermsii* and *B. turicatae*).

§§ Approximately one third of cases included in this summary (35%) were reported to state health departments through laboratory reporting compared with 58% reported by providers, and this proportion did not change over the 10-year period.

¶¶
https://www.cdc.gov/relapsing-fever/prevention/index.html#:~:text%20=%20
